# Fast desensitization of acetylcholine receptors induced by a spider toxin

**DOI:** 10.1080/19336950.2021.1961459

**Published:** 2021-08-10

**Authors:** Na Clara Pan, Tingting Zhang, Shimin Hu, Chunyan Liu, Yuping Wang

**Affiliations:** a Department of Neurology, Xuanwu Hospital, Capital Medical University, Beijing, China; b Beijing Key Laboratory of Neuromodulation, Beijing, China; c Centre of Epilepsy, Beijing Institute for Brain Disorders, Capital Medical University, Beijing, China

**Keywords:** Nicotinic receptor, GsMTX-4, single-channel recording, allosteric desensitization, membrane lipid

## Abstract

Nicotinic acetylcholine receptors (nAChRs) are members of the “cys-loop” ligand-gated ion channel superfamily that play important roles in both the peripheral and central system. At the neuromuscular junction, the endplate current is induced by ACh binding and nAChR activation, and then, the current declines to a small steady state, even though ACh is still bound to the receptors. The kinetics of nAChRs with high affinity for ACh but no measurable ion conductance is called desensitization. This adopted desensitization of nAChR channel currents might be an important mechanism for protecting cells against uncontrolled excitation. This study aimed to show that *Grammostola spatulata* toxin (GsMTx4), which was first purified and characterized from the venom of the tarantula *Grammostola spatulata* (now genus Phixotricus), can facilitate the desensitization of nAChRs in murine C2C12 myotubes. To examine the details, muscle-type nAChRs, which are expressed heterologously in HEK293T cells, were studied. A single channel current was recorded under the cell-attached configuration, and the channel activity (NP_o_) decayed much faster after the addition of GsMTx-4 to the pipette solution. The channel kinetics were further analyzed, and GsMTx-4 affected the channel activity of nAChRs by prolonging the closing time without affecting channel conductance or opening activity. The interaction between nAChRs embedded in the lipid membrane and toxin inserted into the membrane may contribute to the conformational change in the receptor and thus change the channel activity. This new property of GsMTx-4 may lead to a better understanding of the desensitization of ligand-gated channels and disease therapy.

## Introduction

Nicotinic acetylcholine receptors (nAChRs) are heteropentameric ligand-gated ion channels that mediate excitatory neurotransmission at the neuromuscular junction (NMJ) and other peripheral and central synapses. Each muscle-type nAChR consists of two α subunits and one β, δ and γ/ε subunit each in a developmental regulated muscle [1]. A central pore is formed for cation permeation upon ligand binding to the α subunits. At the postsynaptic membrane, nAChRs are clustered at a high density of ~10,000 per μm^2^ [2,3]. This nAChR-centered postsynaptic apparatus is maintained throughout life and is essential for efficacious excitation-contraction coupling at the NMJ. In patients suffering from the acquired autoimmune disease myasthenia gravis, the high density of nAChR is impaired as a result of an autoantibody-induced increase in nAChR turnover and degradation [4]. nAChR subunit mutations, on the other hand, cause congenital myasthenic syndrome [5,6]. Thus, the stability and function of nAChRs are important for NMJ integrity.

The phenomenon of nAChR desensitization was first described in muscle by Katz and Thesleff [7]. At the NMJ, following nAChR-ligand binding due to an increase in ACh concentration, the endplate current rapidly increases and then gradually declines to a low but measurable level. This event begins with two ACh molecules reversibly binding to the nAChR, resulting in conformational changes in channel pore opening. Channel activation can be described by the phenotype of an increasing current. The subsequent decline in current is a manifestation of channel inactivation; with time, ligand-bound nAChRs increasingly adopt desensitized conformations that have a high affinity for ACh but no measurable ion conductance. The low steady-state (ss) current that is maintained in the presence of a high ACh concentration reflects the dynamic equilibrium between ligand-bound-desensitized and ligand-bound-open nAChRs [8,9]. Depending on the methods used, multiple methods of desensitization on a time scale of milliseconds to minutes have been described [7,8,10–13]. The physiological role of nAChR desensitization has been suggested to play a role in synaptic plasticity [14]. Desensitization of the nAChR channel is thought to prevent the overactivation of receptors in pathological conditions and can also lead to a reduction in postsynaptic currents upon repetitive synaptic neurotransmitter release [13]. Thus, this process may serve an important function at the NMJ and has been tuned during the course of natural adaptation.

G. *spatulata* toxin (GsMTx4) was first purified and characterized from the venom of the tarantula *Grammostola spatulata* (now genus Phixotricus) [15]. The 34 amino acid peptide forms a compact structure through three specific disulfide bonds with a net charge of +5 [16]. Structurally, the peptide is a member of the inhibitory cysteine knot peptide superfamily [17] that has been shown to bind to anionic lipids with a higher affinity than to zwitterionic lipids [18,19]. GsMTx-4 has been shown to inhibit several stretch-activated cation channels [20–24].

Here, in addition to inhibiting the mechanosensitivity of muscle-type nAChRs [25], the role of GsMTx-4 in the desensitization of nAChRs was described. Our findings suggested that GsMTx-4 could retain the single channels of nAChRs in a closed-like state rather than an open state through interactions with membrane lipids.

## Materials and methods

### cDNAs and other reagents

Constructs encoding murine nAChR subunits (α, β, γ and δ) were kindly provided by Dr Stanley C. Froehner (University of Washington, Seattle). All expression plasmids were verified by DNA sequencing. GsMTx-4 (toxin from the Chilean Rose Tarantula, *Grammostola spatulata*) was obtained commercially from Peptide Institute, Inc. (Osaka, Japan).

### Cell cultures

Cells from murine C2C12 myoblasts were obtained from the American Type Culture Collection (ATCC, Manassas, VA, USA) and cultured in Dulbecco’s Modified Eagle Medium (DMEM) supplemented with 20% fetal bovine serum (FBS) at 37°C in 5% CO2. Myoblasts were induced to differentiate into myotubes by changing the medium to DMEM containing 2% horse serum (HS). Electrophysiological experiments were performed on myotubes 4–5 days after they were placed in this differentiation medium.

Human embryonic kidney 293 T (HEK293T) cells were cultured in DMEM supplemented with 10% FBS and incubated at 37°C in 5% CO2. One day before transfection, the cells were transferred to 24-well plates and then transfected with cDNAs encoding the four nAChR subunits at the ratio of α: β: γ: δ: GFP = 0.6: 0.3: 0.3: 0.3: 0.1 (μg) per well. When the cells reached 90% confluence, the cells were transiently transfected using Lipofectamine 2000 (Invitrogen). Electrophysiological experiments were performed at 1–2 days after transfection.

### Patch clamp recording

All experiments were performed at room temperature. The recording was conducted with an Axopatch 200B patch-clamp amplifier with associated software (Axon Instruments, Union City, CA). Electrode resistance ranged from 7 to 10 MΩ. Tight seals (>1 GΩ) were obtained on the cell body before the membrane was ruptured with negative pressure. Cell-attached mode was used for single-channel recording, and whole-cell mode was used for drug perfusion recording. For mammalian cell recording, the pipette solution contained 150 mM KCl, 2.5 mM MgCl_2_, 10 mM HEPES, and 0.2 mM EGTA (pH = 7.4), and the bath solution contained 150 mM NaCl, 5 mM KCl, 2 mM CaCl_2_, 1 mM MgCl_2_, 10 mM HEPES and 10 mM glucose (pH = 7.4). The currents were typically digitized at 10 kHz. Macroscopic records were filtered at 2 kHz. Electrophysiological data were analyzed with Clampfit software (version 10.0; Axon Instruments) and Sigmaplot (version 10.0; Systat Software, Inc., Chicago, IL) software. The probability of channel opening was calculated using the equation NP_o_ = T’/T, where T’ is the total open time for a patch over the time T. N is always unknown, so the P_o_ measurement is only as accurate as the estimate of N. Data were acceptable for analysis only for the case, in which the recorded cells showed access resistance (Ra) <30 MΩ and the Rs change <30% throughout the experiment.

### Statistical analysis

The statistical analysis was performed using GraphPad Prism software (GraphPad Prism 8.0, version 8.2.0; GraphPad Software Inc., San Diego, CA, USA). The data are expressed as means ± SEM for Gaussian distribution and median (25%, 75%) for non-Gaussian distribution. The data obtained from different perfusion condition in the same cell were analyzed using one-way analysis of variance (ANOVA) followed by Dunnett’s test. The data obtained from the time-course measurements of single channel NP_o_ were conducted using two-way repeated-measures ANOVA followed by Bonferroni’s post hoc analysis. Probability values of less than 0.05 were considered statistically significant. The data of single channel open time and close time in CTL and GsMTx-4 case were analyzed using Student’s t test. For data exhibiting nonGaussian distribution, a Mann-Whitney U test or a Kruskal-Wallis test were applied.

## Results

### GsMTx-4 accelerates the desensitization of muscle-type nAChRs in C2C12 myotubes

**Figure 1.**
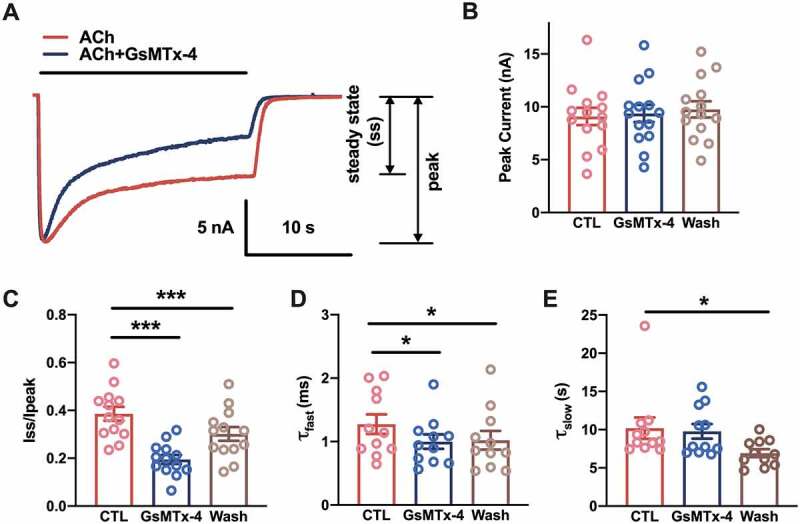
Accelerating desensitization of whole-cell nAChR currents by GsMTx-4 in C2C12 myotubes. (a), Sample traces of whole-cell nAChR currents were generated by the perfusion of 5 µM ACh with/without 1 µM GsMTx-4 in C2C12 myotubes. Holding potential = −70 mV, whole-cell mode. The solid black line indicated the application duration of the ACh or ACh/GsMTx-4. (b), ACh-induced peak current amplitude before (CTL), during (GsMTx-4) and after (Wash) bath perfusion of 1 µM GsMTx-4 (*n* = 11). (c), The ratio of the current of the steady state to the peak amplitude (*n* = 11). D-E, The fast (d) and slow (e) components of the desensitization constant of the whole cell currents described the effect of GsMTx-4 on the rate of nAChR desensitization (*n* = 11). * *p* < 0.05 and ***P < 0.001, one-way ANOVA analysis followed by Dunnett’s test in B-E

Previously, 1 μM GsMTx-4 was used to block the mechanosensitivity of muscle-type nAChRs [25]. During the experiment, toxic inhibition of the nAChR single channel was observed. To determine the properties of GsMTx-4-mediated inhibition, whole-cell currents were recorded in murine C2C12 myotubes. For control condition (CTL), nAChR channels from the recording cell were activated by perfusion of 5 μM ACh until the steady state was reached. After perfusing of the extracellular solution (without ACh) for 20 s, the whole-cell current recovered to the baseline. And then for GsMTx-4 condition (GsMTx-4), 1 μM GsMTx-4 adding 5 μM ACh was perfused to the same cell until the steady state was reached. After at least 1 minute of extracellular solution rinsing, the same cell was activated by 5 μM ACh (Wash condition) until the steady state was reached (Figure 1(a)). The toxin had no effect on the peak currents of nAChRs, which suggested that GsMTx-4 was not an antagonist of nAChRs (Figure 1(b), F_1.524, 15.24_ = 0.452, P = 0.5927). However, one-way ANOVA analysis showed the steady state currents among pre-toxin, during-toxin and post-toxin were significantly changed (Figure 1(c), F_1.885, 18.85_ = 119.9, P < 0.0001). The traces showed that the GsMTx-4-modified currents decreased to a small steady state more than the autologous desensitization of nAChRs (Dunnett’s multiple comparison test: for CTL vs. GsMTx-4, P < 0.0001, for CTL vs. Wash, P < 0.0001). The significant difference between the CTL and Wash might due to the long-lasting extracellular solution rinsing after GsMTx-4 application. Since desensitization is classically portrayed as a two-component phenomenon stemming from the existence of distinct “fast” and “slow” desensitized states [26,27], a two-component decay function was used to fit each whole-cell current of the desensitization of nAChRs. An irreversible decrease in the τ value of the fast phase (τ_fast_) was observed (Figure 1(d), F_1.337, 13.37_ = 6.700, P = 0.0161, Dunnett’s multiple comparison test: for CTL vs. GsMTx-4, P = 0.0326, for CTL vs. Wash, P = 0.0465); however, the slow phase (τ_slow_) showed no change when GsMTx-4 was applied (Figure 1(e), F_1.819, 18.19_ = 6.498, P = 0.0087, Dunnett’s multiple comparison test: for CTL vs. GsMTx-4, P = 0.8837, for CTL vs. Wash, P = 0.0259). These results indicated that GsMTx-4 facilitated fast desensitization but did not block the pore of the nAChR.

**Figure 2.**
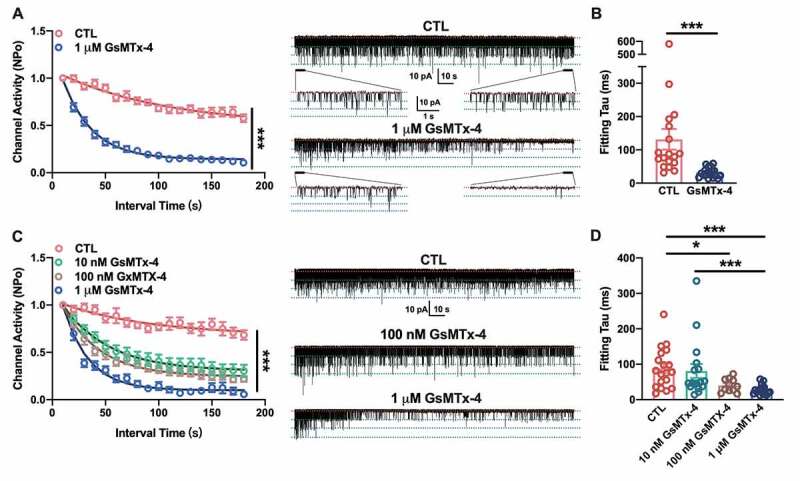
Effect of GsMTx-4 on the single-channel current decay of endogenous nAChRs in C2C12 myotubes and exogenous nAChRs in HEK293T cells. A, The channel activity of the ACh-induced single-channel decay in C2C12 myotubes without (red, *n* = 18) or with (blue, *n* = 19) 1 µM GsMTx-4 in the pipette solution. The curve lines were the fitted results following the function of exponential decay. Right panel: representative traces of 3-min recordings of single channels with magnified 5-s recordings. B, The fitting tau values in (a) under the condition of CTL and 1 µM GsMTx-4. C, The channel activity of ACh-induced single-channel decay in HEK293T cells without toxin (red, *n* = 19), with 10 nM (green, *n* = 17), 100 nM (brown, *n* = 11) or with 1 µM (blue, *n* = 18) GsMTx-4 in the pipette solution. Cells were transfected with the nAChR subunits α, β, δ and γ. The curve lines were fitted results following the function of exponential decay. Right panel: representative traces of 3-min recordings of single channels in transiently transfected HEK293T cells. D, The fitting tau values in (c) under the condition of CTL and different concentrations of GsMTx-4. Pipette ACh concentration was 0.5 µM with a holding potential at +70 mV, cell-attached mode. The red dotted line indicates the closed state of the single channel, and the green dotted line indicates the open state. **P < 0.01 and ***P < 0.001, two-way ANOVA followed by Bonferroni’s test in A and C, Mann-Whitney test in B, and Kruskal-Wallis test in D

Furthermore, acetylcholine-induced single-channel currents were also recorded from C2C12 myotubes using the cell-attached patch-clamp technique. The nAChR single-channel currents were verified by activated under 500 nM Ach in pipette and blocked via 1 μM α-BTX, the specific blocker of nAChR (Supplemental Figure 1(a)). The I–V curve of nAChR single channel current under stepped holding voltage showed an intrinsic channel conductance of nAChR channels (Supplemental Figure 1(b) and 1(c)). To detect the effect of GsMTx-4, the cell-attached recording with the pipette solution containing 500 nM ACh lasted at least 3 min. The single-channel opening showed only a mild decay when there was only ligand in the pipette (Figure 2(a)-right panel, CTL), while the channels opening declined faster when 1 μM GsMTx-4 was added to ACh in the pipette (Figure 2(a)-right panel, GsMTx-4). This effect was quantified by calculating the channel activity of nAChR channels, the NP_o_, in every 10 s interval. Compared with the CTL condition, the GsMTx-4 treatment induced a remarkable decay of single-channel activities (For time and treatment interaction, F_17, 595_ = 16.37, P < 0.0001; for time-lapse, F_7.504, 262.7_ = 120, P < 0.0001; for treatment, F_1, 35_ = 161.8, P < 0.0001; after Bonferroni’s multiple comparison test, P < 0.0001 in each interval point of CTL vs. GsMTX-4). An exponential decay function fitting the decay course showed the constant value of decay, τ in two conditions (Figure 2(b)). For the control, the τ was 83.54 (58.37, 176.2) s, and for the GsMTx-4 condition, median of τ was 29.46 (21.49, 37.91) s (P < 0.0001). This result indicated that GsMTx-4 inhibited the opening ability of nAChR.

### GsMTx-4 decays the activities of single channels of nAChRs in HEK293T

To further verify this effect of GsMTx-4, we expressed nAChRs in HEK293T cells, which do not have endogenous receptors. cDNAs of the muscle-type nAChR subunits α, β, γ and δ were transfected into these cells, which led to the expression of nAChRs on the cell surface. As shown in Figure 2(c), the single-channel currents could be induced by 500 nM ACh in the pipette solution under a cell-attached configuration. However, coexisting of 1 μM GsMTx-4 and 500 nM ACh hastened the single-channel decay of nAChRs. The damping of GsMTx-4 would follow as a dose-dependent manner. The mixed-effects two way ANOVA followed by Bonferroni’s test showed the toxin treatment and time-lapse could be both affect the channel activities prominently (For time and treatment interaction, F_51, 1026_ = 6.390, P < 0.0001; for time-lapse, F_8.301, 501.0_ = 123.1, P < 0.0001; for treatment, F_3, 61_ = 42.26, P < 0.0001; after Bonferroni’s multiple comparison test, P < 0.0001 in each interval point of CTL vs. GsMTX-4 unless labeled as * P < 0.05, ** P < 0.01 and “ns” P > 0.05). However, under the higher concentration of GsMTx-4 (100 nM and 1 μM) the decay constant, τ was significantly smaller than that in CTL (Figure 2(d)). For CTL, τ was 77.61 (36.22, 131.2) s; for 10 nM of GsMTx-4, τ was 47.12 (36.99, 94.80) s; for 100 nM GsMTx-4, τ was 37.18 (25.93, 57.14) s; and for 1 μM of GsMTx-4, τ was 24.48 (17.16, 34.02) s (for CTL vs. 10 nM GsMTx-4, P = 0.3869; for CTL vs. 100 nM GsMTx-4, P = 0.024; for CTL vs. 1 μM GsMTx-4, P < 0.0001; for 10 nM GsMTx-4 vs. 100 nM GsMTx-4, P = 0.1450; for 10 nM GsMTx-4 vs. 1 μM GsMTx-4, P = 0.0008; for 100 nM GsMTx-4 vs. 1 μM GsMTx-4, P = 0.1401). This result indicated that a higher concentration of GsMTx-4 would lead to more effective desensitization of nAChRs.

Additionally, Jahn et al. [28] reported that the desensitization kinetics of nAChR channel currents are the same for embryonic and adult receptor types, with time constants of approximately 50 ms; thus, the effect of GsMTx-4 on the single-channel kinetics of ε-subunit-containing nAChRs had not been tested. In summary, data from endogenous receptors in C2C12 myotubes and exogenously expressed receptors in HEK293T cells showed that GsMTx-4, which was used to block stretch-activated cation channels, could accelerate the desensitization of nAChRs without blocking the opening pore of channels.

### Single-channel kinetics of the effect of GsMTx-4

**Figure 3.**
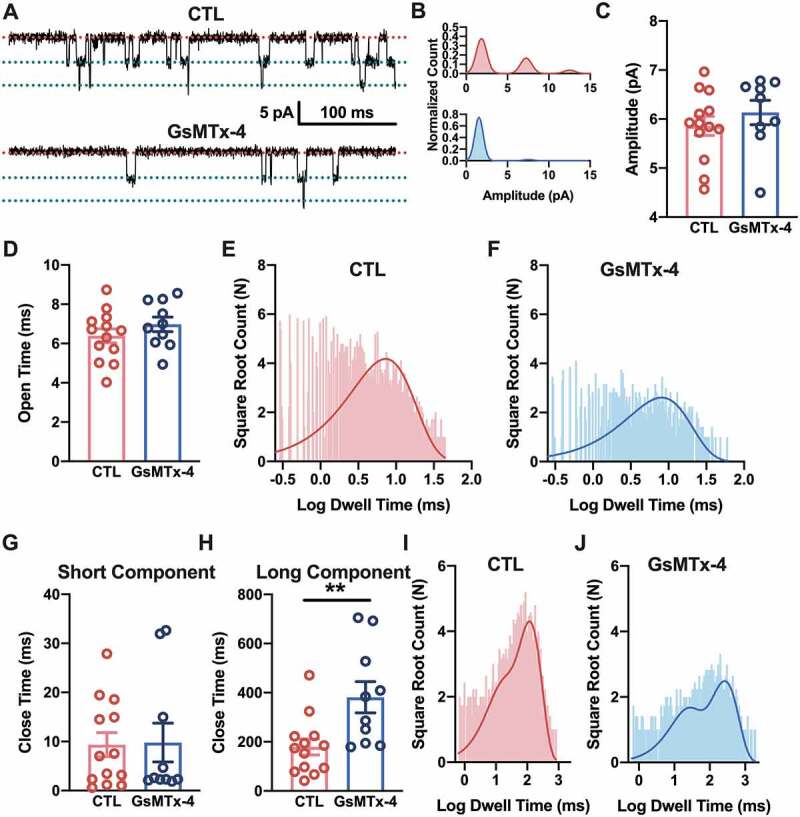
Kinetics of the single channel current of nAChR influenced by GsMTx-4 in HEK293T. A, Detailed ACh-induced single channels in the control and 1 μM GsMTx-4 groups. The red dotted line indicates the closed state of the single channel, and the green dotted line indicates the open state. B, An example of all-point amplitude histogram of the events (upper, control and bottom, GsMTx-4). C, Single-channel current amplitude level (control *n* = 13 and GsMTx-4 *n* = 9) from all patches. D, Dwell times of open events were averaged (for control, *n* = 13 and for GsMTX-4, *n* = 10). E-F, Open dwell time histograms for the control (e) and GsMTx-4 (f) groups. G-H, Dwell close times of the two components of the events (for control, *n* = 13 and for GsMTX-4, *n* = 10). I-J, Closed time histograms for both the control (i) and GsMTx-4 (j) groups. The histograms were fitted (continuous solid line) with an exponential log probability function by automatic comparisons of either single or double components (Clampfit software; Molecular Devices). ** *p* < 0.01, unpaired t-test in C-D and G-H

Although the channel activity was dramatically decayed by GsMTx-4, the amplitude of the single-channel currents of nAChRs were not altered (Figure 3(a)). This is also shown in the histogram of single-channel currents (Figure 3(b)). The amplitude of the first-level single-channel currents was maintained at ~5.8 pA with a pipette holding potential of +70 mV (P = 0.3888, t = 0.8810, df = 20) (Figure 3(c)). It is indicated that GsMTx-4 did not change the structure of the nAChR channel and had no effect on the intrinsic conductance of the channels.

To further characterize effect property of GsMTx-4 on nAChRs, detailed single-channel kinetics were analyzed. Compared with channels only exposed to ACh, the toxin-treated channels had a prolonged closing time but had no significant difference in opening time (Figure 3(d, g) and (h)). Moreover, the open and close times of the channels are shown by the histogram of each channel burst event. For the open time, a single-component fitting by an exponential log probability function showed no obvious difference between the control (Figure 3(e)) and GsMTx-4 (figure 3(f)) groups. The open time in the CTL group was 6.40 ± 0.35 ms, and the open time in the GsMTx-4 group was 6.98 ± 0.37 ms (P = 0.2758, t = 1.119, df = 21). Even more, the instantaneous rates of the channels’ opening for each dose of GsMTx-4 have no significant change (Supplementary Figure 2). For the close time, double-component fitting with the same function, using automatic comparisons, showed that GsMTx-4 (Figure 3(j)) prolonged the duration of channels’ closed state, compared with the duration of channels’ closed state without GsMTx-4 (Figure 3(i)). The close time of the short component of the CTL group was 9.36 ± 2.44 ms with the proportion of 22% versus 9.79 ± 3.95 ms in the GsMTx-4 group with the proportion of 33% (P = 0.9252, t = 0.09505, df = 21), and the close time of the long component of the CTL group was 179.10 ± 32.70 ms with a proportion of 78% versus 381.20 ± 63.65 ms in the GsMTx-4 group with a proportion of 67% (P = 0.0065, t = 3.021, df = 21). These results suggested that GsMTx-4 could maintain channels in the closed state and make them more difficult to open.

## Discussion

Desensitized nAChRs have high-affinity binding sites (open-like) and undetectable ion conduction (closed-like). Current models of nAChR desensitization, like most ligand-gated ion channels, are based on cyclical schemes with two distinct desensitized states: the early, quickly desensitized state and a more slowly inactivated state [11,29,30]. In the case of muscle nAChRs, it is known that there are at least four or five distinct desensitization states [31]. In the presence of a high concentration of ACh (1 mM), the desensitization of nAChRs occurred more rapidly [32]. However, the desensitization induced by 1 mM ACh and GsMTx-4 was quite different because in the presence of an ACh concentration sufficient to cause desensitization, single-channel current pulses occurred in groups with many more channels opening at the same time [30], while 1 μM GsMTx-4 decayed the single-channel current to a much lower measurable level with no change in the number of open channels.

In our study, the spider toxin GsMTx-4 could change single-channel kinetics, even though it maintained the channel in a closed state. Therefore, the fast desensitization of nAChRs may be due to the unique structure of GsMTx-4, which is able to partition into the cell membrane by interacting with nonpolar components, penetrating 0.7–0.9 nm from the lipid center [18,33,34]. It is envisaged that the presence of GsMTx-4 may influence the local composition of membrane lipids, especially, the positively charged group of the GsMTx-4 could interact with carbonyloxygen atoms of the inner leaflet of the plasma membrane [33]. Interestingly, a component of the inner leaflet lipid, PIP_2_ has been verified to interact with the desensitization gate of pentameric ligand-gated ion channels, including nAChRs [35]. The toxin might act as a positive allosteric modulator that modulates the gating process by facilitating nAChR desensitization. Structural changes occurring among the toxin, membrane and receptors may contribute to the desensitization of nAChRs. Further investigation may provide stronger and more detailed evidence of this relationship.

In summary, we propose that GsMTx-4 inserts into membrane lipids and can squeeze the integrative assembly of nAChR subunits. This spatial mechanical effect influences the dynamic kinetics of nAChR single-channels, leading to the accelerated desensitization of nAChRs. Our results complement previous work on the effects of GsMTx-4 on membrane proteins and provide a new insight into the theory of the desensitization of pentameric ligand-gated channels.

## Supplementary Material

Supplemental Material
