# Supplementary figures and images for: Fast desensitization of acetylcholine receptors induced by a spider toxin
Source: Channels (Austin). 2021 Aug 10;15(1):507–15. doi: 10.1080/19336950.2021.1961459 (PMC8366537; doi:10.1080/19336950.2021.1961459)

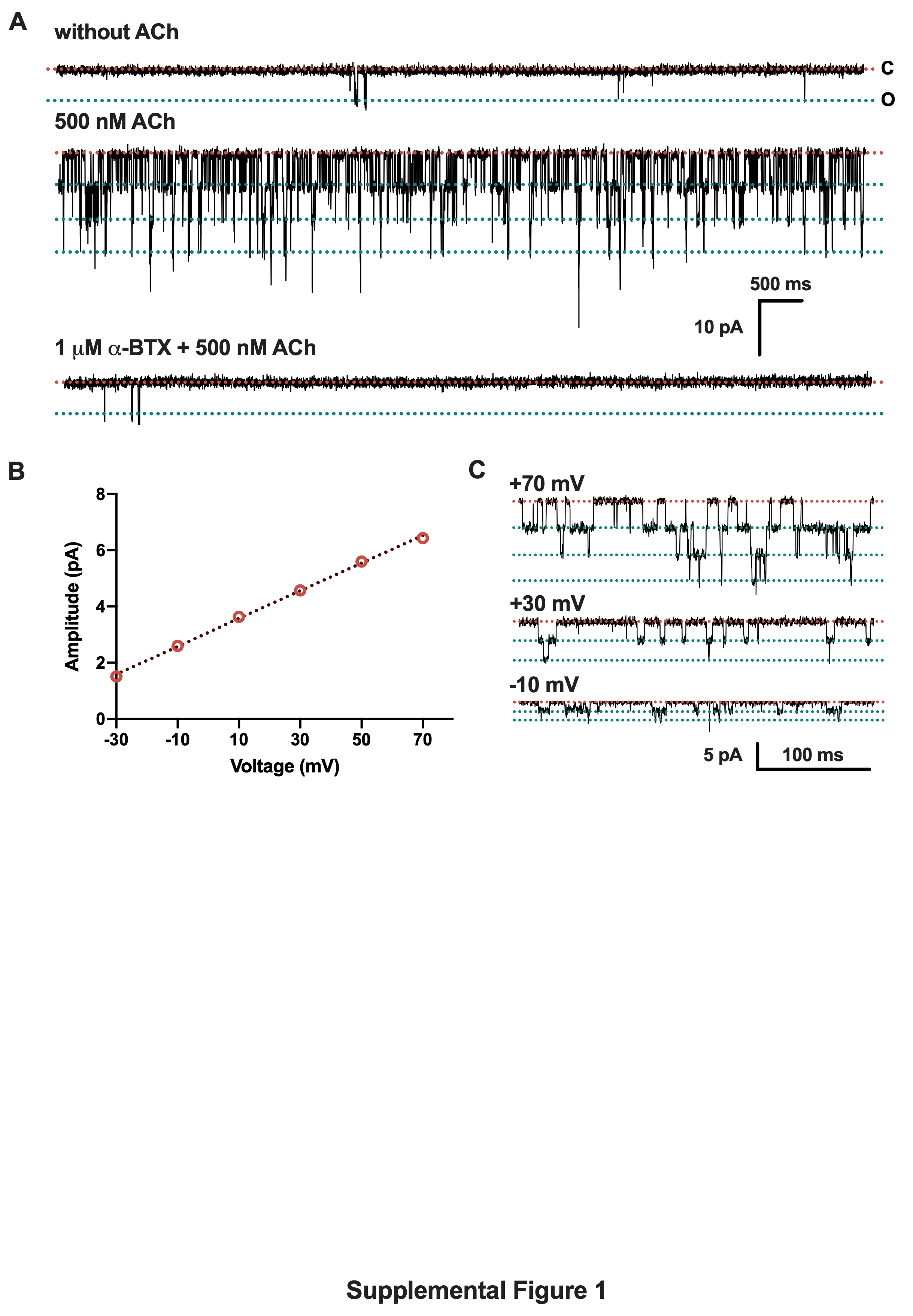

Supplement: Supplemental Material [file KCHL_A_1961459_SM8945.zip › suppl/sup Fig 1.tiff]

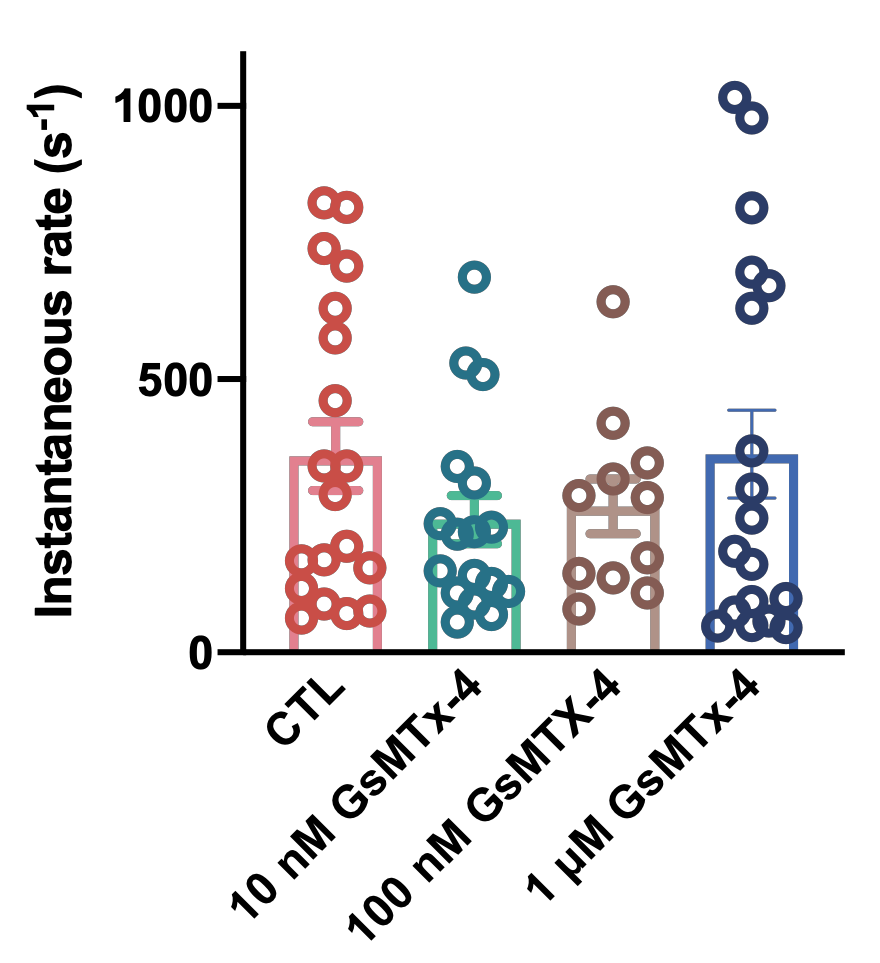

Supplement: Supplemental Material [file KCHL_A_1961459_SM8945.zip › suppl/sup Fig 2.tiff]
